# Efficacy and Safety of Intravenous Thrombolysis in Patients with Acute Ischemic Stroke and Pre–Existing Disability

**DOI:** 10.3390/jcm8030400

**Published:** 2019-03-22

**Authors:** Giovanni Merlino, Elisa Corazza, Simone Lorenzut, Gian Luigi Gigli, Daniela Cargnelutti, Mariarosaria Valente

**Affiliations:** 1 Stroke Unit, Department of Neurosciences, Udine University Hospital, 33100 Udine, Italy; simone.lorenzut@asuiud.sanita.fvg.it (S.L.); daniela.cernelutti@asuiud.sanita.fvg.it (D.C.); 2 Department of Neurosciences, Clinical Neurology, Udine University Hospital, 33100 Udine, Italy; eli.corazza@gmail.com (E.C.); gigli@uniud.it (G.L.G.); valente@uniud.it (M.V.); 3 Neurology Unit, Department of Medicine (DAME), University of Udine, 33100 Udine, Italy; 4 Department of Mathematics, Physics and Informatics (DMIF), University of Udine, 33100 Udine, Italy

**Keywords:** acute ischemic stroke, intravenous thrombolysis, pre-existing disability, modified Rankin Scale, outcomes

## Abstract

Little is known about intravenous thrombolysis (IVT) in acute ischemic stroke (AIS) patients with pre-existing disability. Disabled patients are often excluded from IVT treatment. Previous studies investigated the role of pre-existing disability on outcomes in AIS patients after IVT. However, no studies have been conducted to date to determine whether IVT may improve clinical outcomes in AIS patients with pre-existing disability. The aim of our study was to evaluate efficacy and safety of IVT in patients with pre-existing moderate and moderately severe disability (pre-stroke modified Rankin Scale score = 3 or 4) affected by AIS. This study was based on a retrospective analysis of a prospectively collected database of consecutive patients admitted to the Udine University Hospital with AIS from January 2015 to May 2018. The efficacy endpoints were the rate of favorable outcome and rate of major neurological improvement. The safety endpoints were the rate of mortality at three months, presence of intracranial hemorrhage (ICH), and presence of symptomatic intracranial hemorrhage (sICH). The study population included 110 AIS patients with pre-existing moderate and moderately severe disability, 36 of which received (IVT+) and 74 did not receive IVT (IVT−). AIS disabled patients treated with IVT had higher rates of favorable outcome (66.7% vs. 36.5%, *p* = 0.003) and major neurological improvement (39.4% vs. 17.4%, *p* = 0.01) compared to non-treated ones. Two in three disabled patients returned to their pre-stroke functional status when treated with IVT. Prevalence of three-month mortality, ICH, and sICH did not differ in the two groups. Disabled patients affected by AIS significantly improved after IVT. Moderate and moderately severe disability alone should not be considered, per se, as a contraindication to IVT treatment.

## 1. Introduction

In industrialized countries stroke is the second most important cause of mortality in the world and the third in terms of disease burden, calculated as disability-adjusted life years (DALYs) [1,2]. Indications for use of intravenous thrombolysis (IVT) in patients with acute ischemic stroke (AIS) changed over time and an increasing number of patients could undergo this safe and efficacious treatment [3,4,5]. At this time, little is known about IVT in AIS patients with pre-existing disability.

Patients who could not live alone without daily help of another person prior to stroke, e.g., patients with a modified Rankin Scale (mRS) score ≥3, are often excluded from IVT treatment because of concerns about poor outcomes [6,7,8,9,10]. In fact, these patients account for only 10% of treated cases according to international stroke registries [11,12]. To date, a few studies have been performed in order to evaluate the role of pre-existing disability on outcomes in AIS patients after IVT. The authors observed a higher rate of mortality in functionally dependent patients, while the risk of symptomatic intracranial hemorrhage (sICH) did not increase in these subjects compared to independent patients when treated with IVT [13,14,15,16]. However, no studies have been conducted in AIS patients with pre-existing disability to determine whether IVT may improve clinical outcomes.

The aim of our study was to evaluate efficacy and safety of IVT in patients with pre-existing moderate and moderately severe disability (defined as pre-stroke mRS score = 3 or 4), comparing the clinical outcomes of treated patients with those of the untreated ones.

## 2. Methods

### 2.1. Patients

This study was based on a retrospective analysis of a prospectively collected database of consecutive patients admitted to the Udine University Hospital because of AIS from January 2015 to May 2018. Of 1392 patients admitted for AIS, 144 had a pre-stroke mRS score ≥3 and were suitable for IVT treatment. Since no AIS patients affected by very severe pre-stroke disability (mRS score = 5) had been treated with IVT, we excluded them from the study (*n* = 15). Nineteen additional patients were excluded because of: (1) clinical conditions different from stroke that could have caused fluctuating disability (*n* = 4); (2) treatment with endovascular thrombectomy in addition to IVT (*n* = 8); (3) loss to follow–up (*n* = 7). The remaining sample of 110 AIS patients with a pre-stroke mRS score of 3 or 4 were included in the analysis and were stratified into 2 groups: AIS patients who received IVT (IVT+) and those to whom IVT was denied because of a pre-existing disability (IVT−).

### 2.2. Data Collection

The following variables were collected: Age, sex, vascular risk factors, presence of cognitive impairment, laboratory findings, and pharmacological treatment at admission. Data on causes of pre-stroke dependency were acquired. The functional outcome was assessed by means of the mRS score at admission, based on the pre-stroke disability, and 3 months after the stroke. Stroke severity was determined with the National Institute of Health Stroke Scale (NIHSS) at admission and discharge. The presence of intracranial hemorrhage (ICH) was defined as any parenchymal hematoma based on the European Cooperative Acute Stroke Study (ECASS) morphologic definitions (ECASS PH-1 or PH-2) [17], whereas presence of sICH was based on the ECASS III protocol [4].

### 2.3. Outcome Measures

The efficacy endpoints were: rate of favorable outcome, defined as a return to the pre-stroke mRS 3 months after AIS, and rate of major neurological improvement which was defined as an improvement of ≥8 points on the NIHSS from baseline or a NIHSS score of 0 at discharge. The safety endpoints were: rate of mortality at 3 months, presence of ICH, and presence of sICH.

### 2.4. Statistical Analysis

Baseline characteristics and outcomes of the two patient groups (IVT+ versus IVT−) were compared by means of the chi-square test (Fisher’s exact test) for categorical variables and the Student’s *t*-test for independent samples when the continuous variables had a normal distribution. The Mann-Whitney U test was used when the continuous variables had an abnormal distribution and for ordinal variables. Binary logistic regression was used to explore variables associated with outcome measures. Data were displayed in tables as means and standard deviations (SD), if not otherwise specified. All probability values are two-tailed. A *p* value of <0.05 was considered to be statistically significant. Statistical analysis was carried out using the SPSS Statistics, Version 20.0 for Windows (Chicago, IL, USA).

## 3. Results

Out of 110 AIS patients with pre-existing moderate and moderately severe disability, 36 underwent IVT (IVT+), whereas 74 were not treated with IVT (IVT−). Table 1 reports the baseline characteristics in the two groups. IVT+ patients were older and had more severe strokes at admission compared to the IVT− patients. Prevalence of cognitive impairment was similar between the two groups (IVT+: 41.2% vs. IVT−: 42.9%, *p* = 0.9). Use of antiplatelet therapy at time of admission was significantly more common in the IVT+ patients than in the IVT− group (68.6% vs. 48.6%, *p* = 0.05). Conversely, AIS patients who did not receive IVT were more frequently pretreated with anticoagulants (17.6% vs. 2.9%, *p* = 0.03).

Table 2 reports the causes of pre-stroke disability in IVT+ and IVT− patients.

As reported in Table 3, AIS patients with pre-existing moderate and moderately severe disability had higher rates of favorable outcomes and major neurological improvements when they were treated with IVT. In addition, the prevalence of three-month mortality, ICH and sICH did not differ in the two groups.

**Figure 1:**
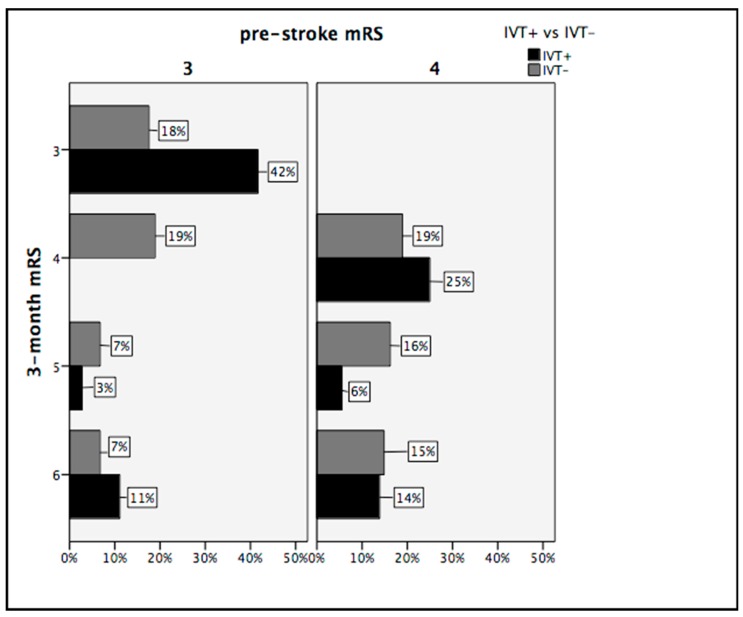
Functional status pre and 3 months after stroke according to intravenous thrombolysis (IVT) treatment on the modified Rankin Scale (mRS).

Figure 1 shows the distribution of mRS scores at admission and three months after the stroke according to IVT treatment.

Significant predictors of efficacy endpoints, other than IVT treatment, by univariate analyses were the following: NIHSS score at admission (OR 0.89, 95% CI 0.84–0.95, *p* = 0.001) for favorable outcome and the serum glucose at admission (OR 0.98, 95% CI 0.97–0.99, *p* = 0.05) for major neurological improvement. Multivariable analyses for efficacy outcomes are shown in Table 4. The statistical models confirmed that use of IVT was an independent predictor of favorable outcome and major neurological improvement in AIS patients with pre-existing disability.

## 4. Discussion

As people continue to live longer and the world’s older population continues to grow at an unprecedented rate [18]. The prevalence of disability increases with age, affecting 40% of elderlies over 60 and 75% of those over 80 years old [19]. Similarly, the incidence of AIS doubles with each decade after the age of 45 years [20]. Since IVT is the standard treatment for patients with AIS, stroke physicians are frequently called upon to make a decision on whether IVT treatment should be used in patients with pre-existing disability. Recent international guidelines suggest that IVT treatment may be reasonable in AIS patients with mRS ≥ 2, however treatment decisions should take into account relevant factors such as their quality of life, social support, and the need for a caregiver [21]. With these social factors in mind, the decision to use IVT for treating AIS patients with pre-existing disability appears to be complex and also raises ethical issues. Presumably, the choice to treat or not to treat with IVT should involve the patients and their families. To date, the use of IVT in disabled patients remains uncommon [11,12].

In 2003, Foell et al. compared the outcomes between patients with and without pre-existing disability undergoing IVT for AIS. Despite a significantly higher mortality rate, disabled patients were able to return to their pre-stroke level of function as often as patients without pre-existing disability [13]. More recently, Karlinski et al. analyzed the data of all patients (*n* = 7250) treated with IVT for AIS between October 2003 and December 2011 that were contributed to the Safe Implementation of Treatment in Stroke-Eastern Europe (SITS-EAST). Similarly to Foell et al., the authors reported that mortality was significantly higher in disabled patients than in those with a pre-stroke mRS score of 0, with the odds ratio increasing with higher mRS scores. Pre-existing disability did not increase the risk of sICH and 1 in 3 previously disabled patients returned to their pre-stroke mRS, thus showing a favorable outcome. The fact that in this study patients with pre-existing moderate or more severe disability represented a very small part of the entire sample of AIS patients receiving IVT (2.4%) confirms that stroke physicians infrequently use recombinant tissue plasminogen activator (rt-PA) in these subjects [14]. Gensicke et al. replicated the results of Karlinski et al. when examining a large sample (*n* = 7430) of IVT-treated stroke patients [15]. In 2018, Zhang et al. showed that following IVT, a similar portion of AIS patients with and without pre-existing disability return to their premorbid functional status following IVT. The authors concluded that IVT should be considered in patients with mild-to-moderate pre-existing disability. However, rt-PA use was uncommon in this sample of AIS patients with moderate and moderately severe pre-stroke disability [16]. Altogether, these studies focused on the role of pre-existing disability on outcomes in AIS patients receiving IVT but did not explore the efficacy and safety of IVT in AIS patients with pre-stroke disability, which was the aim of our study. In fact, we compared disabled patients treated with and without IVT. Because little is known regarding patients with higher mRS score, we decided to include only subjects with moderate and moderately severe disability (mRS score = 3 or 4). In our cohort, the use of rt-PA was associated with increased age and a more severe stroke at admission in disabled patients. Although both these variables are known as independent predictors of poor outcome, IVT+ patients had more than seven times (OR 7.26, 95% CI 2.51–21.02) the odds of favorable outcome and more than three times (OR 3.70, 95% CI 1.32–10.35) the odds of major neurological improvement than IVT− ones. Two in three disabled patients returned to their pre-stroke functional status when treated with IVT. In particular, almost half (42%) of the patients with a pre-stroke mRS score of 3 returned to their pre-existing moderate disability if they received IVT. A similar outcome occurred in only 18% of moderately disabled patients that did not receive rt-PA. Although less prominent, the efficacy of IVT on three-month mRS was also confirmed in patients with moderately severe pre-stroke disability. These results are relevant because a recent population-based, prospective cohort study found that if patients with mild-to-moderate premorbid disability accumulate additional disability as a result of a new ischemic stroke, they had far worse five-year mortality, institutionalization outcomes, and higher five-year health/social-care costs, than those who retained their premorbid disability [22].

IVT treatment and serum glucose at admission were the only two independent predictors of major neurological improvement in our sample of disabled patients. Notably, 39.4% of disabled patients receiving rt-PA showed a significantly improved NIHSS score at discharge. The prevalence of major neurological improvement was greatly lower in IVT− patients.

Previously disabled patients do not seem to be different from the others regarding the better outcome related to onset-to-treatment interval. In fact, a recent study by Nolte et al. showed that prehospital start of IVT treatment on mobile stroke units, as compared with in-hospital care, may translate into better clinical outcomes in patients with pre-stroke dependency [23].

IVT treatment did not increase the risk of three-month mortality in disabled patients. In fact, mortality rate was comparable between IVT+ and IVT− patients, even though subjects in the IVT+ group were significantly older. Only two studies reported the three-month mortality rate in patients receiving IVT with a pre-stroke mRS score of 3–5. Both Karlinski et al. (48.4%) and Gensicke et al. (38.7%) observed a much higher percentage of death with respect to our study (25%) [12,13]. As expected, we observed a trend for ICH and sICH events being more common in patients receiving than in those not receiving IVT. The rate of sICH in our group of disabled IVT+ patients was similar to that reported by Gensicke et al. (5.6% vs. 4.8%), whereas sICH events were observed more frequently by Karlinski et al. (11.7%) [14,15].

We are aware that our investigation was a retrospective observational study with a small sample size. In addition, measures of outcome were obtained by physicians that were not blinded on IVT treatment. Furthermore, the raters of the mRS at three months were not blinded to the pre-stroke mRS, which may have had influenced their rating, and the assessment of the pre-stroke disability with mRS is not completely reliable, due to its intrinsic sensitivity and to interobserver variability [24,25]. Finally, although all patients were qualified for IVT, we were certainly unable to detect all prognostic factors and the baseline differences showed in Table 2 may have missed other unknown intergroup differences, which in theory could have skewed the IVT+ and IVT− population, leading to IVT use only in patients with a better prognosis. However, despite these limitations we demonstrated for the first time that disabled patients affected by AIS significantly improve after IVT. As a consequence, moderate and moderately severe disability alone should not be considered, per se, as a contraindication to IVT treatment. A randomized controlled trial will be needed to validate our results.

## Figures and Tables

**Table 1:** Baseline characteristics.

	IVT+ (*n* = 36)	IVT− (*n* = 74)	*p*
**Demographic data and baseline clinical characteristics**			
Age, years	86.0 ± 8.2	79.6 ± 9.3	0.001
Males, *n* (%)	8 (22.2)	30 (40.5)	0.06
NIHSS score at admission, median (IQR)	10 (5–17.75)	6 (3–15)	0.01
pre-stroke mRS, median (IQR)	3 (3–4)	3.5 (3–4)	0.7
**Vascular risk factors**			
Previous stroke, *n* (%)	5 (13.9)	15 (20.3)	0.4
Atrial fibrillation, *n* (%)	20 (55.6)	35 (47.3)	0.4
Hypertension, *n* (%)	29 (80.6)	63 (85.1)	0.5
Diabetes mellitus, *n* (%)	9 (25.0)	25 (33.8)	0.3
Hypercholesterolemia, *n* (%)	12 (33.3)	30 (41.1)	0.4
Current smoking, *n* (%)	1 (4.8)	5 (10.6)	0.6
**Laboratory findings**			
Serum glucose, mg/dL	139.6 ± 59.0	126.4 ± 60.8	0.3
Creatinine, mg/dL	1.12 ± 0.6	1.01 ± 0.5	0.3
aPTT ratio	1.01 ± 0.1	1.15 ± 0.6	0.1

IVT = intravenous thrombolysis; NIHSS = National Institute of Health Stroke Scale; mRS = modified Rankin Scale; aPTT = activated Partial Thromboplastin Time; IQR = Interquartile Range.

**Table 2:** Causes of pre-stroke disability.

	IVT+ (*n* = 36)	IVT− (*n* = 74)	*p*
**Medical conditions**			0.7
Multifactiorial, *n* (%)	12 (33.3)	23 (31.3)	
Dementia, *n* (%)	7 (19.4)	12 (16.2)	
Degenerative or traumatic bone diseases, *n* (%)	6 (16.7)	8 (10.8)	
Heart diseases, *n* (%)	4 (11.1)	9 (12.2)	
Cerebrovascular diseases, *n* (%)	4 (11.1)	8 (10.8)	
Cancer, *n* (%)	0 (0)	7 (9.5)	
Other neurological disorders, *n* (%)	1 (2.8)	4 (5.4)	
Other causes, *n* (%)	2 (5.6)	3 (4.1)	

**Table 3:** Efficacy and safety endpoints.

	IVT+ (*n* = 36)	IVT− (*n* = 74)	*p*
**Efficacy endpoints**			
Favorable outcome, *n* (%)	24 (66.7)	27 (36.5)	0.003
Major neurological improvement, *n* (%)	13 (39.4)	12 (17.4)	0.01
**Safety endpoints**			
Mortality at 3 months, *n* (%)	9 (25)	16 (21.6)	0.6
ICH, *n* (%)	4 (11.1)	4 (5.5)	0.4
sICH, *n* (%)	2 (5.6)	0 (0)	0.1

IVT = intravenous thrombolysis; ICH = Intracranial Hemorrhage; sICH = symptomatic Intracranial Hemorrhage.

**Table 4:** Multivariable analyses showing the independent predictors of favorable outcome and major neurological improvement.

**Favorable outcome**	**OR**	**95% CI**	***p***
IVT treatment			
No	1.00	2.51–21.02	0.001
Yes	7.26		
NIHSS at admission	0.87	0.81–0.93	0.001
**Major neurological improvement**	**OR**	**95% CI**	***p***
IVT treatment			
No	1.00	1.32–10.35	0.01
Yes	3.70		
Serum glucose at admission	0.98	0.97–0.99	0.04
